# Matrix Metalloprotease 9 Mediates Neutrophil Migration into the Airways in Response to Influenza Virus-Induced Toll-Like Receptor Signaling

**DOI:** 10.1371/journal.ppat.1002641

**Published:** 2012-04-05

**Authors:** Linda M. Bradley, Mia F. Douglass, Dhrubamitra Chatterjee, Shizuo Akira, Bas J. G. Baaten

**Affiliations:** 1 Infectious and Inflammatory Disease Center, Sanford-Burnham Medical Research Institute, La Jolla, California, United States of America; 2 Laboratory of Host Defense, World Premier International Immunology Frontier Research Center, and Department of Host Defense, Research Institute for Microbial Diseases, Osaka University, Osaka, Japan; University of Wisconsin-Madison, United States of America

## Abstract

The early inflammatory response to influenza virus infection contributes to severe lung disease and continues to pose a serious threat to human health. The mechanisms by which neutrophils gain entry to the respiratory tract and their role during pathogenesis remain unclear. Here, we report that neutrophils significantly contributed to morbidity in a pathological mouse model of influenza virus infection. Using extensive immunohistochemistry, bone marrow transfers, and depletion studies, we identified neutrophils as the predominant pulmonary cellular source of the gelatinase matrix metalloprotease (MMP) 9, which is capable of digesting the extracellular matrix. Furthermore, infection of MMP9-deficient mice showed that MMP9 was functionally required for neutrophil migration and control of viral replication in the respiratory tract. Although MMP9 release was toll-like receptor (TLR) signaling-dependent, MyD88-mediated signals in non-hematopoietic cells, rather than neutrophil TLRs themselves, were important for neutrophil migration. These results were extended using multiplex analyses of inflammatory mediators to show that neutrophil chemotactic factor, CCL3, and TNFα were reduced in the *Myd88* ^−/−^ airways. Furthermore, TNFα induced MMP9 secretion by neutrophils and blocking TNFα *in vivo* reduced neutrophil recruitment after infection. Innate recognition of influenza virus therefore provides the mechanisms to induce recruitment of neutrophils through chemokines and to enable their motility within the tissue via MMP9-mediated cleavage of the basement membrane. Our results demonstrate a previously unknown contribution of MMP9 to influenza virus pathogenesis by mediating excessive neutrophil migration into the respiratory tract in response to viral replication that could be exploited for therapeutic purposes.

## Author Summary

Influenza-associated morbidity and mortality due to yearly epidemics and sporadic, devastating pandemics are a significant health and economic burden. Severe complications arising from highly virulent viruses are associated with rapid, massive inflammatory cell infiltration. Although neutrophils are the predominant cell population recruited to the lung in response to pandemic influenza viruses, the mechanisms by which they gain entry to the respiratory tract remain unclear. In this study, we show a previously unknown contribution of MMP9 to influenza pathogenesis by mediating excessive neutrophil migration into the lung, which not only controls viral replication, but also contributes to morbidity. The *in vivo* relevance of MMP9-derived enzymatic activity in neutrophils is controversial and understudied, but our data provide new evidence that innate recognition of influenza virus attracts neutrophils that secrete MMP9, which enables them to traverse the basement membrane of the lung by digesting the extracellular matrix. The dichotomy of MMP9 function in immunity versus pathology provides real challenges for targeting MMP9 for therapeutic purposes. Nevertheless, finding the balance to modulate neutrophil numbers following influenza virus infection will allow for innate immunity to be boosted whilst preventing pathology associated with pandemic strains.

## Introduction

Influenza viruses are highly contagious and cause an average of 226,000 hospitalizations and 36,000 deaths in yearly epidemics [1]. The emergence of immunologically distinct influenza viruses to which a whole population is susceptible has resulted in sporadic, devastating pandemics, such as the recent outbreak of ‘swine flu’ that infected an estimated 61 million people and killed 12,470 [2]. Furthermore, there is growing concern that avian influenza viruses, which have a significant mortality rate, could mutate to allow easy human-to-human transmission [3]. Severe complications arising from pandemic influenza or highly pathogenic avian viruses are often associated with rapid, massive inflammatory cell infiltration [4]. Inflammatory cytokines and chemokines have been associated as their dysregulation correlates strongly with high viral load and pathology [5]. Following infection, influenza virus replication occurs in the epithelial cells of the respiratory tract where innate immune responses are initiated by recognition of the virus through the inflammasome, which consists of toll-like receptors (TLRs), retinoic acid-inducible gene-I-like receptors, and NOD-like receptors [6]. For example, viral double-stranded RNA and single-stranded RNA are recognized by TLR3 and TLR7, respectively [7], [8]. TLR3/7 signaling induces an inflammatory response that promotes a cascade of immune processes that regulate cellular recruitment and function, including the induction of cytokines and chemokines. Whereas this process results in full maturation of dendritic cells (DCs), activation and recruitment of antigen-specific T cells, and adequate anti-influenza humoral responses [9], [10], aberrant signaling has been suggested to play a key role in mediating lung pathology that is characterized by excess inflammation and pulmonary destruction [4].

MMPs are a family of proteolytic enzymes that are involved in remodeling the extracellular matrix (ECM) under both physiological and pathological conditions [11]. They can be produced by a range of cells in the respiratory tract, where they mediate wound healing, airway remodeling, and cell trafficking [12]. As such, MMPs play an important role in immunity, and their proteolytic activity can also directly dampen the inflammatory potential by downregulating cytokine and chemokine function [13]. However, excessive responses may contribute to pathology and MMP activity has been implicated in a variety of pulmonary diseases. Since MMPs have the potential to cause significant host damage, their production and function are tightly regulated. For example, MMPs are rarely stored inside cells and require gene transcription before secretion, the exceptions being neutrophil MMP-8 and -9, which are constitutively expressed in granules. Active MMPs can degrade all components of the ECM and are divided into subclasses based on their substrate specificity. Collectively known as the gelatinases, MMP2 and MMP9 cleave denatured collagens (gelatins) and type IV collagen present in basement membranes [13]. Although implicated in a range of pulmonary diseases, a detailed understanding of the involvement of gelatinases in disease pathology after viral infection is lacking. MMP9 is not expressed in healthy lungs, but may be released under inflammatory conditions (such as infections and neoplastic diseases) by macrophages, mast cells, lymphocytes, and neutrophils. Although neutrophils are the predominant leukocyte population to be recruited to the lung and appear early in the immune response to pandemic influenza viruses [14], the mechanisms by which they gain entry to the respiratory tract and their role during pathogenesis remain unclear.

In this study, we sought to identify the role of MMP9 in the recruitment of neutrophils into the respiratory tract after influenza virus infection. By increasing the infective dose, we were able to mimic many of the hallmarks of more virulent “pandemic” infections by virtue of inducing increased morbidity, inflammation, and lung pathology. High dose infection led to increased neutrophil recruitment to the lungs and airways, which contributed significantly to morbidity. We identified neutrophils as the predominant source of pulmonary MMP9. Importantly, MMP9 was functionally required for neutrophil migration into the lung in response to infection and for control of viral replication. Although MMP9 release from neutrophils was TLR-dependent, MyD88-mediated signals in non-hematopoietic cells, rather than intrinsic induction, were important for neutrophil recruitment by inducing the neutrophil chemoattractant CCL3. In addition, MyD88 signaling-induced tumor necrosis factor (TNF)α contributed to MMP9 release from neutrophils. Our data demonstrate a previously unknown role of MMP9 to influenza virus pathogenesis by contributing to excessive neutrophil migration into the respiratory tract in response to TLR-induced chemotactic factors.

## Results

### Morbidity, inflammation, and neutrophil number are increased after infection with high dose influenza virus

**Figure 1:**
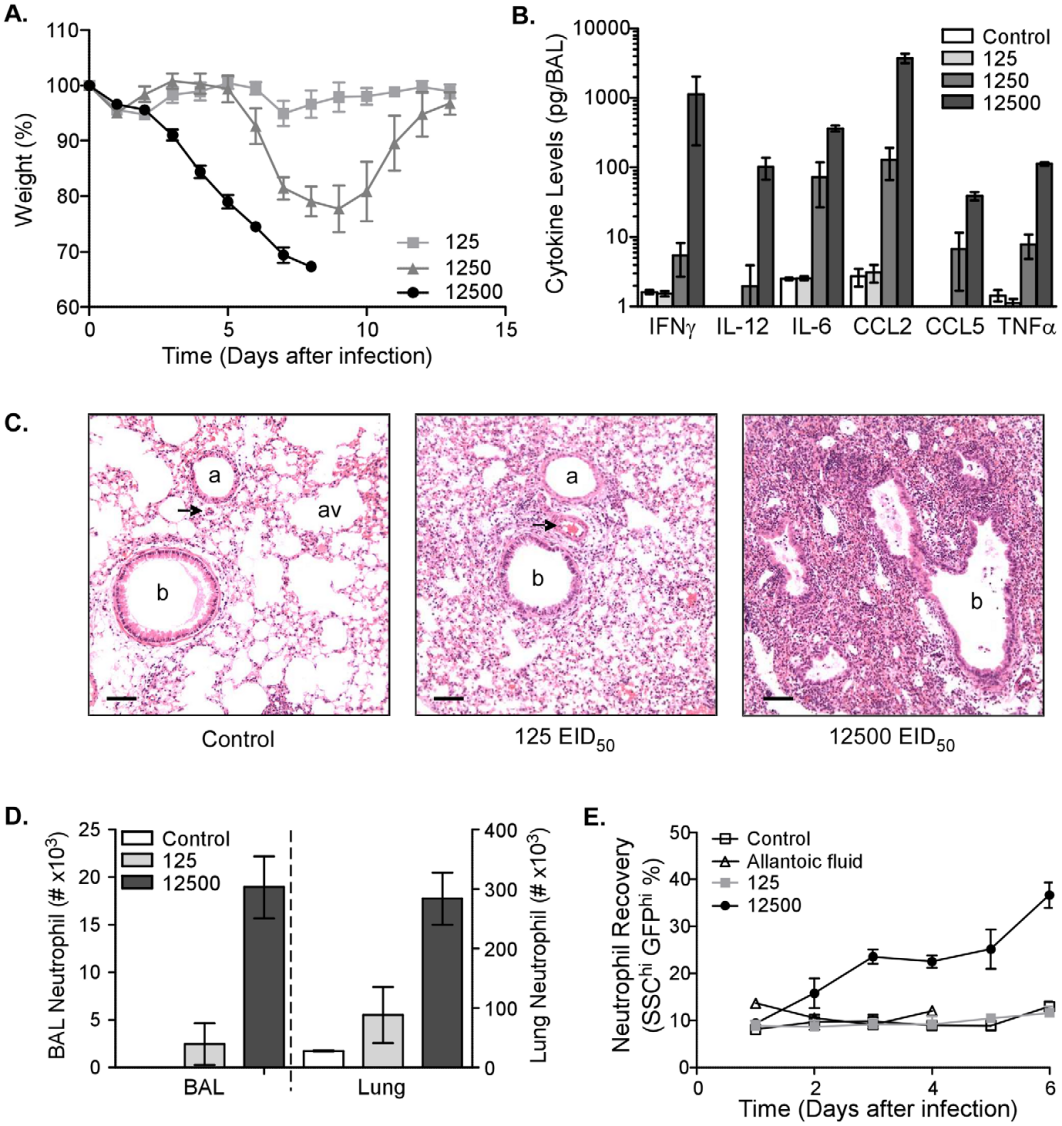
Morbidity, inflammation, and neutrophil number are increased after infection with high dose influenza virus. (A) Kinetics of weight loss as a percentage of starting weight in C57BL/6 mice infected i.n. with 125 (*light-grey squares*), 1250 (*dark-grey triangles*), or 12500 (*black circles*) EID_50_ PR8 virus. Mean ± SEM (n = 5, representative of four independent experiments). (B) Cytokine and chemokine levels in the BAL of uninfected mice (‘control’, *clear bars*) or mice infected 6 days earlier with 125 (*light-grey bars*), 1250 (*grey bars*), or 12500 (*dark-grey bars*) EID_50_ PR8 virus. Mean ± SEM (n = 3–4, representative of two independent experiments). (C) Histological examination of uninfected lungs (control, *left*) or lungs 6 days after infection with 125 (*middle*) or 12500 (*right*) EID_50_ PR8 virus. Perfused lungs were fixed in formalin and stained for hematoxylin and eosin (scalebar = 100 µm). Images are representative of multiple mice. b. bronchiole, a. arteriole, av. alveole, and arrowhead. venule. (D) Neutrophil numbers in the BAL and lung of uninfected mice (‘control’, *clear bars*) or mice infected 6 days earlier with 125 (*light-grey bars*) or 12500 (*dark-grey bars*) EID_50_ PR8 virus. Mean ± SEM (n = 3, representative of two independent experiments). (E) The percentage of blood neutrophils from LysM-GFP mice infected with 125 (*light-grey squares*) or 12500 (*black circles*) EID_50_ PR8 virus compared to control (PBS, *open squares*) or allantoic fluid at the same dilution as the highest viral dose (*open triangles*). Mean ± SEM (n = 4–5).

Infection of humans with virulent viruses is associated with high viral titers and increased inflammation [5]. To determine the effect of infective dose on cytokine/chemokine levels and cellular recruitment, mice were infected with different doses of Influenza virus A PR/8/34 (PR8): 125, 1250, or 12500 50% egg infectious doses (EID_50_). Morbidity, measured by weight loss, correlated with the infective dose (Figure 1A). Mice began losing weight as early as 3 days after infection with 12500 EID_50_ and progressively lost more until they had to be sacrificed in accordance with animal welfare guidelines. Although delayed, mice receiving the intermediate dose (1250 EID_50_) lost weight, but recovered with the appearance of adaptive immunity. Depending on the infective dose, mice had increased cytokine/chemokine levels in the airways 6 days after infection as measured by multiplex analysis of the broncheo-alveolar lavage (BAL) (Figure 1B). Inflammatory cytokines, such as IFNγ, interleukin (IL)-6 and TNFα, and the chemokines, CCL2 and CCL5, were highly upregulated in the respiratory tract after infection with 12500 EID_50_. The increased secretion of inflammatory mediators was associated with increased cellular infiltrates in the lung 6 days after infection (Figure 1C). Whereas uninfected lungs had clear airspaces, cellular infiltrates and bronchoconstriction were observed after infection with 125 EID_50_. Increasing the dose tenfold resulted in complete obliteration of the normal lung architecture in many foci of infiltration (Figure 1C, *far right*). The increased cellularity of the lung was in part made up by a substantial number of infiltrating neutrophils (Figure 1D). Analysis of lung and BAL by flow cytometry demonstrated a significant increase in the number of Ly6G+ cells in both sites after infection with 12500 EID_50_. Other cell populations that increased after infection included macrophages, CD4 and CD8 T cells (data not shown). To address further neutrophil recruitment, we utilized LysM-GFP mice, which express enhanced green fluorescent protein (GFP) from the lysozyme M gene locus, as a reporter for neutrophil numbers in the blood [15]. An increased percentage of circulating neutrophils (GFP^hi^ side-scatter^hi^) could be detected as early as two days after infection, but only with the higher dose of virus (Figure 1E). Their percentage kept increasing until mice had to be sacrificed 6 days after infection. In contrast, neutrophil numbers in LysM-GFP mice infected with 125 EID_50_ did not increase and were similar to uninfected control mice or those given allantoic fluid. Thus, by using a high dose of infectious virus, we established a model to induce severe inflammation and increased neutrophil accumulation in the respiratory tract.

### MMP9 is produced by neutrophils after infection

**Figure 2:**
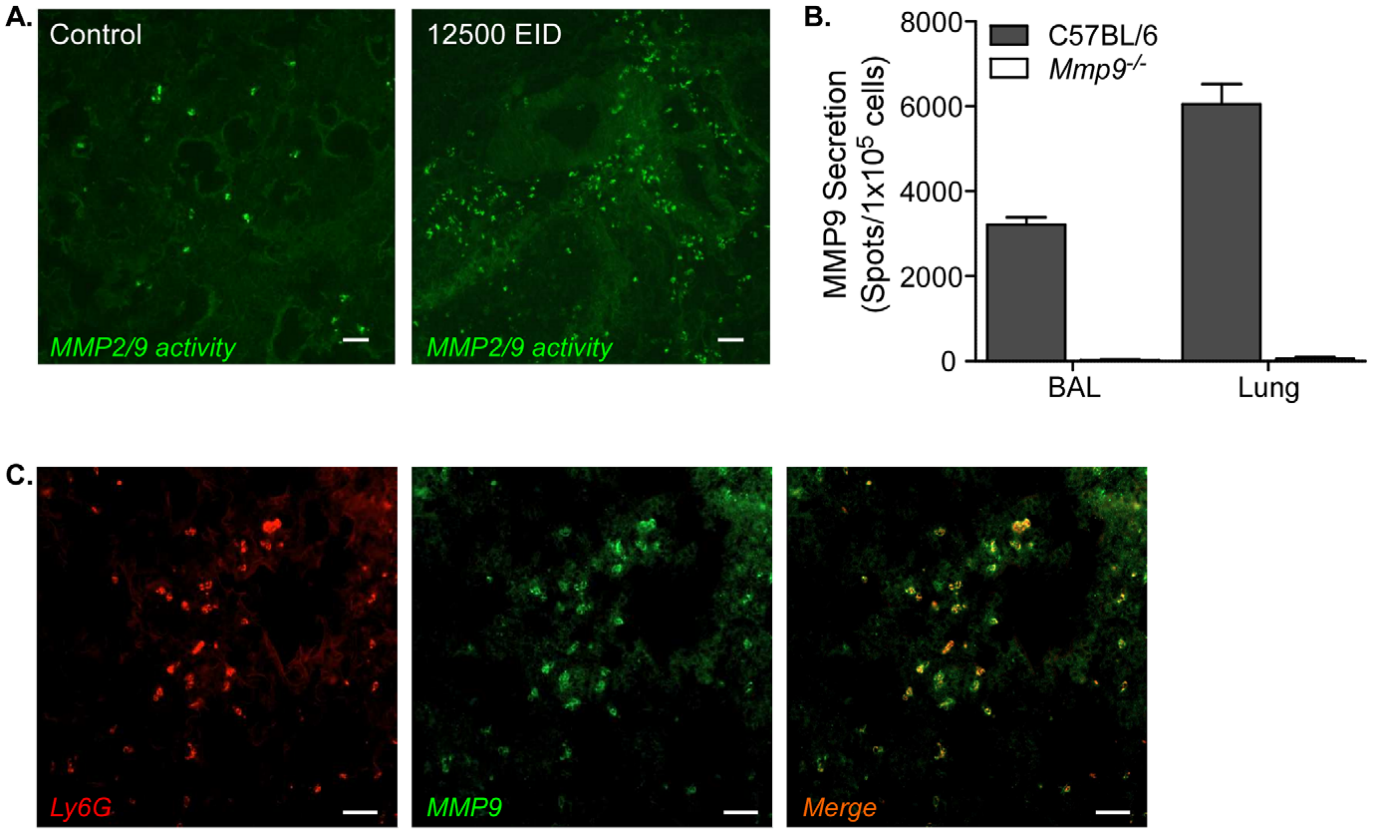
MMP9 is produced by neutrophils after infection. (A) MMP2/9 activity in lungs of uninfected control mice (*left panel*) or those 6 days after infection (*right panel*). Gelatinase activity was measured by *in situ* zymogram and visualized by green fluorescence after enzymatic cleavage to release fluorochrome from a quencher (scalebar = 50 µm). Images are representative of multiple mice. (B) MMP9 secretion by cells from BAL and lung from Ly5.1 recipient mice that received BM from either *Mmp9* ^−/−^ (*clear bars*) or C57BL/6 (*grey bars*) donors was measured by ELISPOT. Mean ± SEM (n = 5–7, representative of two independent experiments). (C) Ly6G+ lung neutrophils (*red*) and MMP9+ cells (*green*), and their colocalization (*merge, right panel*) were visualized by immunofluorescence of infected lung 6 days after infection (scalebar = 50 µm). Images are representative of multiple mice.

Our model allowed us to investigate the contribution of MMPs to pathogenesis. Gelatinases MMP2 and MMP9 are capable of cleaving type IV collagen present in the basement membrane and have been implicated in a variety of pulmonary diseases [13]. We assessed total gelatinase activity in lung tissue by *in situ* zymography 6 days after influenza virus infection (Figure 2A). Enzymatic cleavage of the probe was significantly increased in the mice infected with 12500 EID_50_ and seemed to be restricted to defined areas, rather than displaying generalized activity throughout the lung. MMP9 expression correlated with the original infective dose (data not shown) and appeared to be expressed in the vicinity of infected cells, which was visualized using double-staining for MMP9 and viral hemaglutinin (HA) (Figure S1). Of note was the expression of MMP9 in between the airways and foci of viral replication (Figure S1B).

MMP9 can be produced in many cell types and to identify a potential candidate cell population, we transferred C57BL/6 or *Mmp9* ^−/−^ bone marrow into wild-type recipients and allowed the mice to reconstitute for at least four weeks (tested by flow cytometry, data not shown). Mice were infected with 12500 EID_50_ virus and MMP9 secretion was assessed in the airways (BAL) and lung parenchyma by ELISPOT 6 days later (Figure 2B). Mice reconstituted with C57BL/6 bone marrow had significantly more MMP9-secreting cells in both the airways and lung. However, virtually no MMP9 secretion could be observed when the hematopoietic system was deficient in *mmp9*, suggesting that bone marrow-derived cells produced the majority of MMP9 in the lung after infection.

To identify the cells that produce MMP9, we did extensive dual immunofluorescence analysis of lung tissues and found Ly6G+ colocalization with MMP9 (Figure 2C). Unlike the Gr-1 marker, Ly6G is specifically expressed on neutrophils, but not on plasmacytoid DC, CCR2+ inflammatory monocytes, or myeloid suppressor monocytes [16]. Thus, we identified neutrophils as the predominant cellular source of MMP9 produced after influenza virus infection.

### Depletion of neutrophils during influenza virus infection abrogates MMP9 secretion

**Figure 3:**
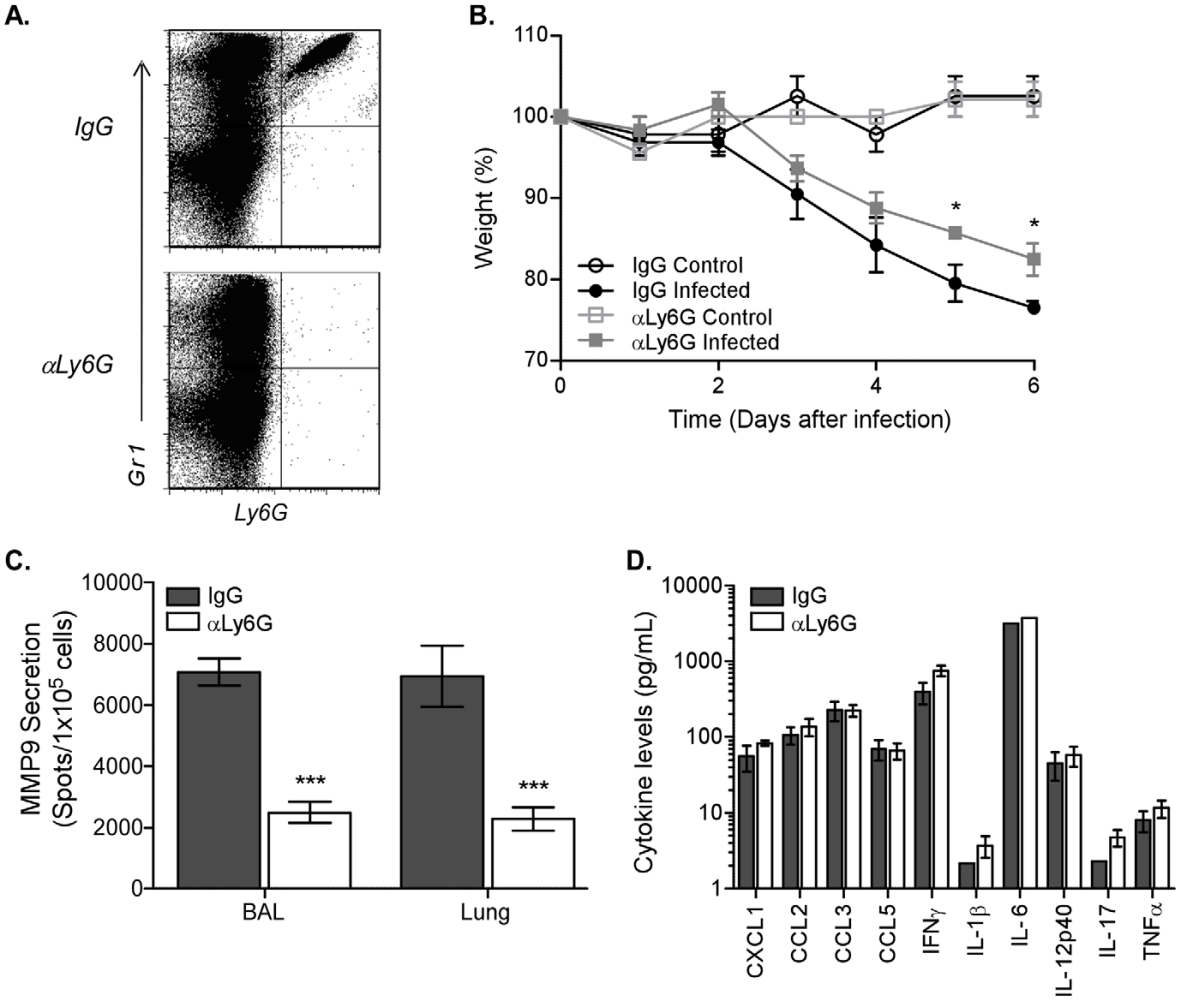
Depletion of neutrophils abrogates MMP9 secretion after influenza virus infection. Neutrophils were depleted by injecting C57BL/6 mice with 400 µg anti-Ly6G antibody (αLy6G) or isotype control (IgG) one day before infection and every other day thereafter. (A) Depletion of Ly6G+ cells in αLy6G-treated C57BL/6 mice (*bottom panel*) compared to IgG (*top panel*) was verified by flow cytometric analysis 6 days after infection. (B) Kinetics of weight loss as a percentage of starting weight in uninfected (control, *open symbols*) and infected (*closed symbols*) mice treated with the αLy6G (*squares*) or IgG isotype (*circles*). (C) MMP9 secretion by cells from BAL and lung from infected mice that were treated with αLy6G (*clear bars*) or IgG isotype (*grey bars*) was measured by ELISPOT. (D) Inflammatory cytokine release in airways after neutrophil depletion. BALs were collected 6 days after infection and supernatants assayed by bead array. (B–D) Mean ± SEM (n = 3, representative of two independent experiments). (B–C) Considered significant at *P<0.05, ***P<0.001.

Since neutrophil numbers were significantly increased following high dose infection and this population appeared to be the predominant source of MMP9 in the lung after infection, we sought to identify the direct function of MMP9 in this cell population. Ly6G is exclusively expressed on neutrophils and antibody treatment has been shown before to specifically deplete neutrophils only [17]. Treating C57BL/6 mice one day before infection and every two days thereafter with an antibody to Ly6G completely depleted Ly6G+ neutrophils as compared to the isotype control IgG (Figure 3A). Importantly, in the absence of neutrophils, there was a significant reduction in weight loss at 5 and 6 days after pathogenic influenza virus infection (Figure 3B). Our data suggest that neutrophils were pathogenic after high dose infection and contribute to morbidity. Furthermore, we demonstrated that MMP9 secretion correlated with morbidity, since neutrophil depletion also significantly reduced MMP9 exocytosis (Figure 3C), confirming our results that MMP9 was predominantly produced by neutrophils after influenza virus infection (Figure 2C).

In addition to containing MMP9 in preloaded granules, neutrophils are capable of secreting a variety of inflammatory cytokines or chemokines [18], which could be modulating the MMP9 response after infection indirectly. Therefore, the levels of innate mediators in the BAL were tested 6 days after infection by bead array analysis. The concentrations of the tested inflammatory cytokines and chemokines were similar between the anti-Ly6G and isotype-treated mice (Figure 3D), excluding a direct effect of these neutrophil-derived soluble mediators in influenza virus pathogenesis. Although a recent study demonstrated that neutrophils enhanced the response of virus-specific CD8 T cells [19], we did not observe differences in IFNγ production by CD8 T cells between the antibody-treated groups in our model (Figure S2). Thus, our data demonstrate that Ly6G+ neutrophils are capable of producing MMP9 and do not affect influenza virus pathogenesis through production of inflammatory mediators or by indirectly affecting CD8 T cell function.

### Neutrophils require MMP9 to migrate to the respiratory tract

**Figure 4:**
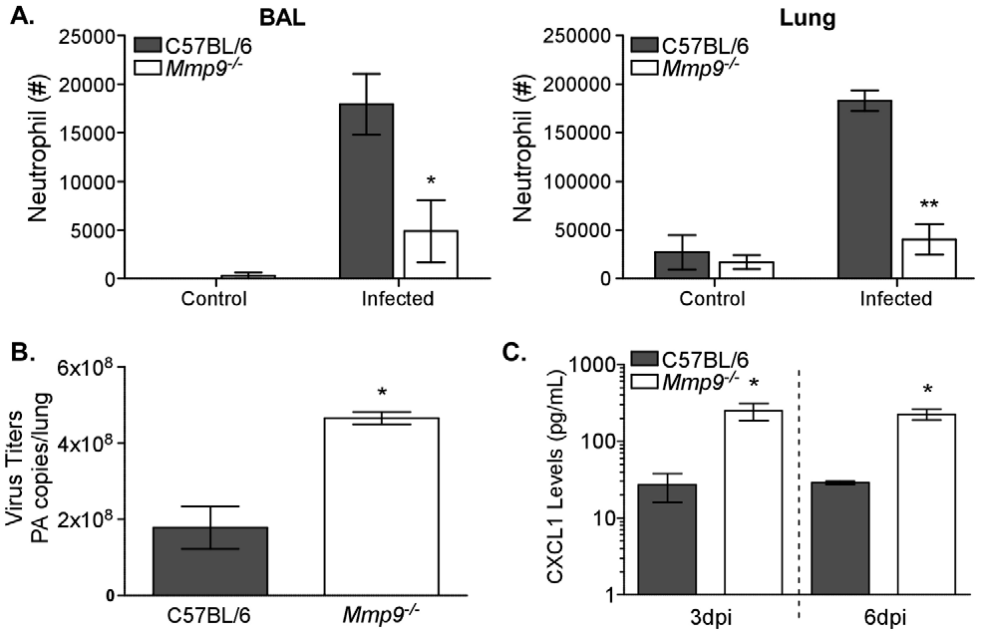
Neutrophils require MMP9 to migrate to the respiratory tract. (A) Neutrophil numbers in the BAL (*left panel*) and lung (*right panel*) were enumerated by flow cytometry 3 days after infection of C57BL/6 mice or *Mmp9* ^−/−^ mice. Control mice were not infected. Mean recovery numbers per lung ± SEM (n = 3–4, representative of three independent experiments). (B) Viral load was enumerated in left lung lobe by quantitative RT-PCR for the influenza PA gene 3 days after infection. Mean PA copies/lung ± SEM (n = 4). (C) Chemokine levels in airways of *Mmp9* ^−/−^ mice after infection. BALs were collected 3 and 6 days after infection and supernatants assayed for CXCL1 by bead array. Mean ± SEM (n = 4, representative of two independent experiments). (A–C) Considered significant at *P<0.05, **P<0.01.

Unlike the chemokines, our results implicate MMP9 in neutrophil-mediated morbidity. Although controversial, proteolytic digestion of the basement membrane lining the lung endothelium has been proposed to be able to mediate migration of cells [20]. To address the functional role of MMP9 during influenza virus pathogenesis, we infected C57BL/6 and *Mmp9* ^−/−^ mice and quantified the number of neutrophils in the respiratory tract. Ly6G+ cells were significantly decreased in both BAL and lung from the *Mmp9* ^−/−^ mice 6 days after infection (Figure 4A). As a consequence, the viral load in the lungs of the *Mmp9* ^−/−^ mice was significantly increased, demonstrating that MMP9, and possibly neutrophils, are required for viral clearance (Figure 4B). Airway levels of CXCL1, CCL2, CCL3, CCL5, IFNγ, IL-6, and TNFα were measured 3 and 6 days after infection. We could only identify significant increases in CXCL1 both days (Figure 4C and Figure S4). Recovery of alveolar macrophages (F4/80^hi^CD11c^hi^) and exudate macrophages (F4/80^int^CD11c^int^), which could contribute to the inflammatory response, were not significantly different 3 days after infection of *Mmp9* ^−/−^ mice compared to C57BL/6 controls (Figure S3A). Likewise, lung pathology was not impacted in *Mmp9* ^−/−^ mice at this time point (data not shown). These results support the notion that MMP9 is crucial for neutrophil, but not macrophage, migration to the infected lung and thereby not only contributes to influenza virus pathogenesis, but is also required for control of viral replication.

### Neutrophil recruitment, MMP9 secretion, and viral load are TLR-dependent

**Figure 5:**
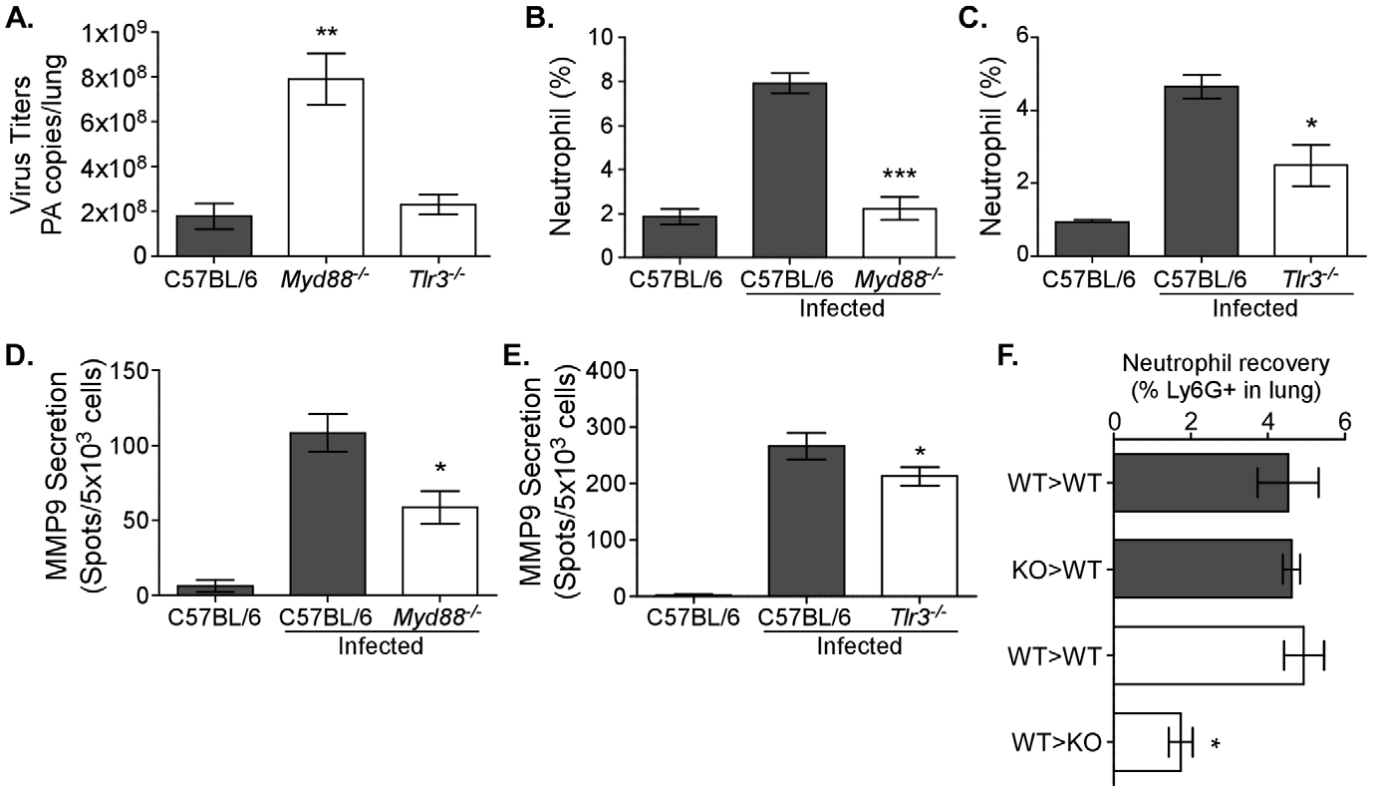
Neutrophil recruitment, MMP9 secretion, and viral load are TLR-dependent. Neutrophil numbers and MMP9 secretion are decreased in TLR-deficient mice. (A–E) C57BL/6 (*grey bars*), *Myd88* ^−/−^, and *Tlr3* ^−/−^ (*both clear bars*) mice were infected with 12500 EID_50_ PR8. (A) The viral load was enumerated in left lung lobes of C57BL/6, *Myd88* ^−/−^, and *Tlr3* ^−/−^ mice by quantitative RT-PCR for the influenza PA gene 3 days after infection. Mean PA copies/lung ± SEM (n = 4, representative of two independent experiments). (B, C) The percentage of neutrophils in lungs of *Myd88* ^−/−^ and *Tlr3* ^−/−^ mice, respectively, were enumerated by flow cytometry 6 days after infection. (D, E) The number of cells secreting MMP9 in the airways of mice deficient in TLR signaling. MMP9 ELISPOT analysis of BALs from (D) *Myd88* ^−/−^ or (E) *Tlr3* ^−/−^ mice. Mean ± SEM (n = 4–5, representative of two independent experiments). (F) Neutrophil recoveries in lung 3 days after infection of mice receiving bone marrow transfer. The percentage of neutrophils after transfer of C57BL/6 (WT>WT) or *Myd88* ^−/−^ (KO>WT) bone marrow into Ly5.1 congenic mice (*grey bars*) or after transfer of Ly5.1 bone marrow cells into Ly5.2+ C57BL/6 (WT>WT) or *Myd88* ^−/−^ (WT>KO) recipients (*clear bars*) is shown. (A–E) Mean ± SEM (n = 3, representative of two independent experiments). (A–F) Considered significant at *P<0.05, **P<0.01, ***P<0.001.

We next investigated the mechanism(s) responsible for MMP9-mediated neutrophil migration. Since influenza-associated inflammation is initiated and dependent on TLRs [7], [8], we inquired if TLR signaling was also involved in MMP9-mediated neutrophil recruitment. To this end, mice deficient in the TLR adaptor molecule MyD88 (mediates all TLR signaling, except for that by TLR3) and *Tlr3* ^−/−^ mice were infected with 12500 EID_50_ and the effect on viral replication, MMP9 secretion, and neutrophil numbers was assessed. Morbidity in both strains was not significantly different (data not shown). Furthermore, overall lung pathology was only marginally ameliorated in *Myd88* ^−/−^ mice 3 days after infection (data not shown). However, viral titers were significantly increased in the lungs of *Myd88* ^−/−^ mice (p<0.01), but not *Tlr3* ^−/−^ mice 3 days after infection (Figure 5A). When cellularity was examined by flow cytometry, the percentage of neutrophils in the lungs was significantly reduced in *Myd88* ^−/−^ (p<0.001) and *Tlr3* ^−/−^ (p<0.05) mice (Figure 5B–C). In accordance, MMP9 secretion was also significantly reduced in the airways in both strains (Figure 5D–E). In contrast, recovery of exudate macrophages from the airways of *Myd88* ^−/−^ or *Tlr3* ^−/−^ mice compared to C57BL/6 controls was similar 3 days after infection (Figure S3B–C). However, whereas alveolar macrophage recovery was unaffected in *Myd88* ^−/−^ mice, there was a significant decrease in their percentage in the *Tlr3* ^−/−^ mice. These results highlight a role for MyD88-derived signals in MMP9 production from neutrophils.

TLRs are expressed on neutrophils [18], [21] and following ligation could induce activation. An inability to recognize the virus may therefore result in decreased MMP9 granule exocytosis. However, it was unclear from our approach whether MMP9 secretion is impaired due to the absence of TLRs in/on the neutrophils themselves or if the absence of MyD88 signaling in other cells affected neutrophil migration, thereby indirectly reducing MMP9 levels in the *Myd88* ^−/−^ airways. In order to distinguish the contribution of MyD88 signaling by immune cells versus non-hematopoietic cells, two bone marrow transfer approaches were used. In the first, bone marrow cells from C57BL/6 or *Myd88* ^−/−^ mice were transferred into Ly5.1 recipients (WT>WT and knock-out (KO)>WT, respectively). Upon reconstitution, all groups were infected with 12500 EID_50_ and 3 days later the number of neutrophils was enumerated by flow cytometry. No differences could be observed in the lungs (Figure 5F) or BALs (data not shown) of mice receiving wildtype or *Myd88* ^−/−^ bone marrow, suggesting that MyD88 signaling in neutrophils themselves is not involved. In the second set of experiments, the inverse approach was performed by transferring congenically marked wildtype cells (Ly5.1) into either C57BL/6 or *Myd88* ^−/−^ recipients (WT>WT and WT>KO, respectively). In these experiments, a reduction in the number of neutrophils gaining access to the respiratory tract of the *Myd88* ^−/−^ recipients was observed (Figure 5F). Interestingly, the data suggest that MyD88 signaling in non-hematopoietic cells is responsible for neutrophil migration into the lung, rather than TLR recognition affecting neutrophil MMP9 secretion directly.

### Neutrophil chemotactic factors in the airways are MyD88-dependent

**Figure 6:**
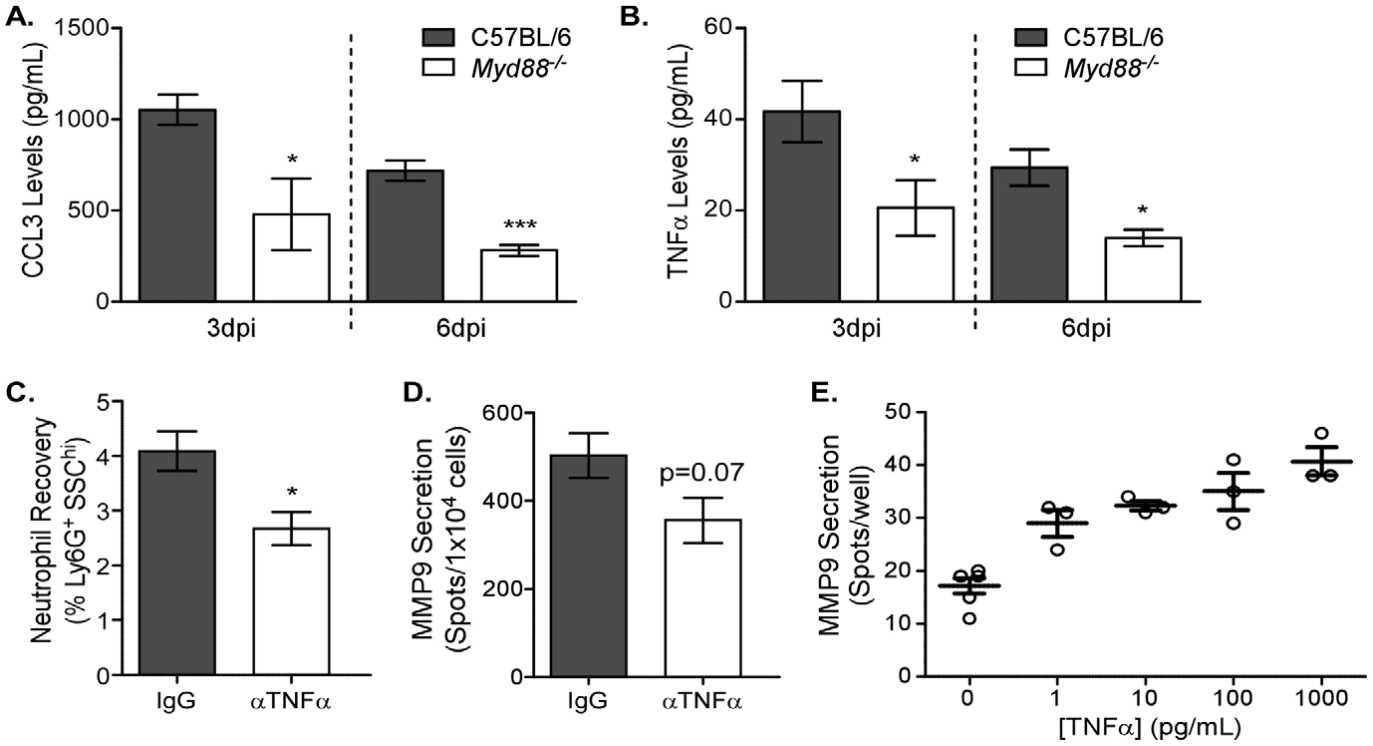
MyD88-dependent TNFα expression induces MMP9 in neutrophils. (A–B) BALs of C57BL/6 (*grey bars*) and *Myd88* ^−/−^ (*clear bars*) mice infected 3 or 6 days earlier were analyzed by bead array. Airway levels of (A) CCL3 and (B) TNFα. Mean ± SEM (n = 3, representative of two independent experiments). (C–D) TNFα function was blocked by injecting C57BL/6 mice with 200 µg anti-TNFα (αTNFα, *clear bars*) or IgG isotype (IgG, *grey bars*) daily starting one day before infection. (C) The percentage of neutrophils in lungs of antibody-treated mice was enumerated using flow cytometry 3 days after infection. (D) The number of cells secreting MMP9 in the lungs was assessed by ELISPOT 3 days after infection. Mean ± SEM (n = 5, representative of two independent experiments). (E) Neutrophils were negatively enriched from the bone marrow of C57BL/6 mice and stimulated with different doses of recombinant TNFα for 6 hours. MMP9 expression during the 6 hour incubation was assessed by ELISPOT (n = 3, representative of two independent experiments). (A–C) Considered significant at *P<0.05, **P<0.01, ***P<0.001.

The indirect nature of MyD88-mediated neutrophil recruitment suggests that chemotaxis to the respiratory tract could be affected. To assess whether reduced cytokine or chemokine expression contributed to the decreased neutrophil accumulation, levels of soluble mediators in the BALs of *Myd88* ^−/−^ mice were analyzed 3 dpi (Figure 6). CCL2, CCL5, IFNγ, IL-1β, IL-6, and IL-17 were not different between wildtype and genetically deficient mice (data not shown). However, in keeping with the reduced neutrophil levels, CCL3 and TNFα levels were reduced in the airways of *Myd88* ^−/−^ mice. The role of chemoattractant CCL3 has been described in neutrophil recruitment and suggested that influenza virus recognition via TLRs in the lung induces chemotactic factors that facilitate neutrophil migration into the infected lung.

We further wanted to investigate the role of TNFα in MMP9-mediated neutrophil recruitment. *In vivo* anti-TNFα antibody administration of C57BL/6 mice one day before infection and every day thereafter resulted in a significant reduction in neutrophil recruitment 3 days after administration of 12500 EID_50_ PR8 (Figure 6C). Furthermore, MMP9 secretion tended to be lower in the lung following anti-TNFα antibody treatment (p = 0.07) (Figure 6D). Since the effect of TNFα on neutrophil recruitment *in vivo* may be multifactorial (e.g. differential adhesion receptor expression on the endothelium), the direct consequence of TNFα on enriched neutrophils was tested *in vitro*. Negatively enriched neutrophils were stimulated with different doses of recombinant TNFα and MMP9 secretion tested by ELISPOT (Figure 6E). A dose-dependent increase in MMP9 secretion was observed, highlighting the direct role of TNFα in regulating MMP9 release from neutrophils. Altogether these data demonstrate a mechanistic link between influenza virus-induced MyD88 signaling in non-hematopoietic pulmonary cells to facilitate neutrophil recruitment by inducing chemotactic and proteolytic mediators needed for their motility *in situ*.

## Discussion

Although implicated in a variety of pulmonary diseases, most notably asthma and tuberculosis, a detailed understanding of the involvement of gelatinases in lung disease pathology is lacking. In this study, we addressed the contribution of MMP9 during influenza virus pathogenesis, which had been implicated recently by virtue of increased concentrations in the serum of patients with influenza and elevated enzymatic activity in mouse lung homogenates [22], [23]. However, the cellular origin and direct role of MMPs in the pathogenesis of influenza virus have not been previously examined. Thus, we focused on elucidating the effects of MMP9 in a pathological mouse model and identified neutrophils as the predominant pulmonary source of MMP9. We demonstrated that MMP9 mediated neutrophil migration into the infected respiratory tract and that it was required for viral clearance. Although MMP9 release was TLR signaling-dependent, MyD88-mediated signals in non-hematopoietic cells, rather than neutrophil TLRs themselves, were important for neutrophil recruitment. This is most likely due to MyD88-controlled induction of the known neutrophil chemoattractant, CCL3, as well as TNFα, which we found induced MMP9 secretion in neutrophils. Our data demonstrate a previously unknown role of MMP9 to influenza virus pathogenesis by contribution to excessive neutrophil migration into the respiratory tract.

Using a model wherein a lethal dose of virus mimics many of the characteristics observed after pandemic infections, with greater cytokine/chemokine levels, lung pathology, and inflammatory cells [5], [14], we showed that MMP9 activity was upregulated in infected lungs, confirming data published recently [23], [24]. We extended these studies by demonstrating that MMP9 was exclusively produced by hematopoietic cells. It is surprising that non-hematopoietic cells did not contribute to MMP9 secretion, as it can be induced under inflammatory conditions in lung epithelial and endothelial cells [25]. Nevertheless, our data further indicated that MMP9 was predominantly secreted in the lung by neutrophils. Neutrophil depletion caused a significant reduction in the number of cells being able to produce MMP9, but did not abrogate all MMP9 production, suggesting that other immune cells can also contribute. In a recent study, neutrophil depletion did not affect MMP9 levels in the BAL 5 days after sublethal influenza virus infection, which is at odds with our results [24]. However, contributing factors to this discrepancy most likely involve the timing, which was later, and the infective dose, which was lower. Nevertheless, excessive neutrophil infiltration into the lung correlated with increased MMP9 production and viral replication when macrophages were depleted [24], which supports our findings. However, the source of MMP9 was not identified. We are currently investigating the potential role of other cell populations that are capable of producing MMP9 under inflammatory conditions, such as lymphocytes [13]. However, unlike these cells, neutrophils do not require *de novo* generation of MMP9 and instead are preloaded in the bone marrow and exocytose their granules following stimulation. In addition, they can synthesize and secrete cytokines and chemokines [18], although we were unable to demonstrate a significant contribution of this mechanism to airway inflammation, as depletion of neutrophils did not cause a significant difference in an extensive panel of potential candidates.

While neutrophils are a major cell population recruited to the lung early in the immune response to pandemic influenza viruses [14], [26], their role during pathogenesis remains unclear. Depletion studies demonstrated enhanced susceptibility of mice to infection, exacerbated inflammation, and increased viral titers and mortality rates [24], [26], [27], [28], which suggested a critical contribution of neutrophils to innate immunity. In contrast, other studies parallel ours and show that neutrophils contribute to disease during severe influenza virus infection [29], [30], [31]. The disparate results may be a consequence of differential infective doses and genetic background. Nevertheless, we show for the first time that MMP9 may contribute to pathogenesis by mediating excessive neutrophil migration into the respiratory tract in response to viral infection. While depletion of neutrophils reduced morbidity, MMP9 was still required for viral clearance. It is likely that the same mechanism(s) that contribute to immunity cause pathology in a more virulent setting [4]. Neutrophils are able to inhibit virus replication *in vitro* and the release of anti-viral molecules by neutrophils could contribute to viral clearance from the lung [24], [27], [32]. However, the mechanisms that allow for neutrophil influx in response to uncontrolled or high viral load, such as MMP9 proteolytic activity, may exacerbate pathology due to collateral destruction of the lung ECM. This could explain the apparent contradicting reports on the role of neutrophils in viral infections and suggests that, while they contribute to viral clearance, excessive numbers contribute to pathology.

We showed that MMP9 functions by regulating neutrophil localization to the respiratory tract. Cellular influx into the airways is a complex process whereby cells cross the endothelial cell layer, the basement membrane, and the epithelial cell layer. Although rolling and tethering on the endothelium has been described in detail, the means by which cells further progress through the basement membrane to reach the airways remain unclear [33]. Whereas gelatinase activity has been implicated in neutrophil migration through artificial basement membranes *in vitro*, its relevance *in vivo* remains controversial [20]. Chemokine-induced migration of neutrophils in an induced lung inflammation model was demonstrated to be independent of MMP9 [34], whereas that in a dermal model did require MMP9 [35]. In part, this discrepancy may be due to the artificial nature of the experiments: while chemokines may be able to mimic inflammation and attract cells, crucial factors necessary for the release of MMP9 may not be available as they are not induced by the chemokine itself (see below). The presence of these required accessory molecules is assured in the influenza model, but at the same time can be difficult to define. Nevertheless, we were able to identify at least one, TNFα, which was able to induce MMP9. It is still of great interest to identify other factors during influenza virus infection as they have obvious clinical relevance. Thus, our data not only demonstrate a role for MMP9 in influenza pathogenesis, but also provide further *in vivo* evidence for MMP9 activity as a mechanism by which neutrophils digest the extracellular matrix to traverse the basement membrane in order to gain access to the infected lung epithelium. The colocalization of MMP9 and viral antigen and its expression between the endothelium and areas of viral replication support this hypothesis. In addition to its role in mediating proteolytic cleavage of the basement membrane to allow for motility within the lung, MMP9 may also modulate chemokine expression itself by digesting CXCL-1 [13]. Our finding that CXCL-1, a potent neutrophil chemoattractant, is significantly upregulated in the *Mmp9* ^−/−^ mice following infection hints at a negative feedback loop in which MMP9 release by neutrophils disrupts the CXCL-1 gradient and dampens further neutrophil recruitment in the absence of influenza virus.

We hypothesized that TLRs are important to induce MMP9 secretion from neutrophils. These innate pattern recognition receptors are expressed by neutrophils [18]. Furthermore, viral RNA has been detected in neutrophils [36], which could facilitate access to TLRs inside endosomes, such as TLR3 and TLR7, which have both been shown to be able to recognize influenza virus. Indeed, TLR7 signaling was demonstrated to be able to activate neutrophils in response to influenza virus, although this was dependent on GM-CSF [21]. Therefore, we examined the role of TLR signaling in neutrophil activation in *Tlr3* ^−/−^ and *Myd88* ^−/−^ mice. MyD88 is the adapter molecule for all known TLRs with the exception of TLR3, and the inclusion of both strains therefore encompassed all potential TLR-mediated effects. Although both neutrophil accumulation and MMP9 secretion were reduced following high dose influenza virus infection, our bone marrow chimera approach demonstrated that this was not a direct effect as *Myd88* ^−/−^ neutrophils still migrated into the respiratory tract. Furthermore, *in vitro* stimulation with influenza virus of neutrophils enriched from *Myd88* ^−/−^ mice did not affect MMP9 secretion (data not shown). Rather, *Myd88* ^−/−^ signaling in non-hematopoietic cells was important for neutrophil accumulation. When we examined chemokine levels in the airways of *Myd88* ^−/−^ mice, CCL3, a known neutrophil chemoattractant, was downregulated. Thus, it appears that rather than a direct effect on MMP9 activation, ablation of MyD88 signaling may have affected the chemokine gradient that would normally attract the neutrophils to the sites of viral replication. In addition, TNFα induced the release of MMP9 from neutrophils and antibody blocking of TNFα prevented their recruitment into the lungs in response to influenza virus infection *in vivo*. TNFα has been demonstrated previously to induce MMP9 exocytosis in a protein kinase C-dependent fashion [37] and we hypothesize that the reduced levels in the *Myd88* ^−/−^ airways prevented MMP9 release and resulted in reduced neutrophil numbers.

The role of neutrophils in viral infections has often been overlooked, on the pretext that they are mostly involved in the clearance of bacterial and fungal pathogens. Their contribution to influenza virus-induced pathology is unclear, but we showed in our pathological model that they contributed to morbidity. Furthermore, we demonstrated that the mechanism for neutrophil-mediated migration to the respiratory tract in response to influenza required MMP9 and depended on extrinsic TLR-signaling. Collectively, our results suggest that innate sensing of viral infection results in a MyD88-dependent induction of chemotactic factors that induce the recruitment of neutrophils. Following diapedesis into the lung parenchyma, MyD88-induced TNFα in non-hematopoietic cells induces MMP9 release in neutrophils and MMP9 enzymatic activity is utilized by these cells to degrade the basement membrane and facilitate their motility within the lung. These novel findings are probably not restricted to influenza pathogenesis, but may also have implications for other viral infections. The dichotomy of the necessity for MMP9-mediated immune cell migration and its role in immunopathology provides real challenges for targeting MMP9 for therapeutic purposes after influenza virus infection. Nevertheless, finding the balance to modulate neutrophils allows for innate immunity to pandemic strains to be boosted whilst preventing potential pathology.

## Materials and Methods

### Ethics statement

All experiments in this study were approved by the Institutional Animal Care and Use Committee (IACUC) at the Sanford-Burnham Medical Research Institute and were carried out in strict accordance with the recommendations in the Guide for the Care and Use of Laboratory Animals of the National Institutes of Health.

### Mice and infection

Male C57BL/6J (C57BL/6J ) mice and B6.SJL-Ptprca Pep3/BoyJ (Ly5.1) were purchased from Jackson Laboratories, USA. *Myd88* ^−/−^ [38], *Tlr3* ^−/−^ [39], *Mmp9* ^−/−^ [40], or LysM-GFP [15] mice (all on C57BL/6 background) were obtained with permission as gifts from Drs S. Swain (University of Massachusetts, MA), M. Corr (University of California, San Diego, CA), F. Kherradmand (Baylor College of Medicine, TX), and G. Srikrishna (Sanford-Burnham Medical Research Institute, CA), respectively. All mice were used at 6–16 weeks of age and held under specific-pathogen-free conditions in the vivarium at the Sanford-Burnham Medical Research Institute.

Influenza virus A PR/8/34 (PR8, H1N1) was grown in the allantoic fluid of 10-day-old embryonated eggs (McIntyre Poultry, San Diego) and stored at −80°C until use. Before infection, mice were anesthetized by intraperitoneal injection of 100 µL ketamine/xylazine (14.3 mg/mL Ketaset (Fort Dodge, IA)/2.86 mg/mL Anased (Lloyd Laboratories, IA)). Mice were infected with 1.25×10^4^ EID_50_ influenza virus (unless otherwise stated) in 50 µL by the intranasal route. Mice were terminated at the indicated timepoints or when clinical signs reached pre-defined endpoints (body weight loss of 30%) by intraperitoneal injection of avertin (2% (w/v) 2,2,2-tribromoethanol, 2% (v/v) tert-amyl-alcohol) or CO_2_ inhalation.

### Preparation of BAL and lung cell suspensions

Cells from the airways and lung were obtained from infected mice at the indicated timepoints. Cells from airways were obtained via BAL by piercing the diaphragm, transecting the trachea, and infusing the lungs with 1 mL PBS through an 18-gauge catheter (Terumo, USA). The resulting cell suspension was centrifuged (5 min, 300×g), the supernatants aspirated and stored at −80°C for cytokine bead array (see below), and the cells resuspended in wash medium (HBSS (CellGro, USA) supplemented with 1% (v/v) heat-inactivated fetal calf serum, 5 mM HEPES (CellGro), and 100 U/mL Pencillin, 100 µg/mL Streptomycin, 292 µg/mL L-glutamine (Mediatech)). Perfused lungs were cut into small pieces, digested in 2 mL collagenase (1 mg/mL collagenase D (Roche, USA), 0.5% bovine serum albumin (BSA, Sigma), 100 µg/mL Dnase) for 60 min at 37°C, and filtered and homogenized over a 70 µm cell sieve. Cell suspensions were washed and resuspended in cell medium and the cell numbers enumerated.

### Cytokine bead array

Cytokine production in the airways after infection was measured using a Legend-plex custom cytokine/chemokine array according to manufacturer's instructions (Biolegend, USA). Supernatants aspirated from BAL were assayed for indicated combinations of the following analytes: IL-1β, IL-6, IL-12p40, IL-17a, IFNγ, CXCL1, CCL2, CCL3, CCL5, and TNFα. Samples were analyzed on a Luminex IS200.

### Flow cytometry

The percentage of neutrophils present in the airways or lung after infection was analyzed by flow cytometry. Recovered cells were stained with monoclonal anti-mouse antibodies Ly6G-APC (1A8) and Gr1-PE (RB6–8C5) (Biolegend). Cells were stained for 15 min on ice before being washed and analyzed on a FACSCalibur or LSR2 Fortessa flow cytometer (Becton Dickinson, CA) using FlowJo 8.8.6 (Tree Star, Ashland, USA). The number of neutrophils in each tissue was calculated from the cell recovery and the percentage positive by flow cytometry.

### Histochemistry, immunostaining, and *in situ* gelatinase assay

For microscopic analysis of pathology, lung tissues were fixed in buffered formaldehyde-saline (pH 7.5), embedded in paraffin, and sectioned. Each lung was stained with H&E prior to examination. Tissues sections for immunofluorescence staining were cut (8 µm) using a cryostat (Leica Jung CM3000), air-dried overnight, fixed in acetone for 10 min, and stored at −35°C. Endogenous biotin in sections was blocked (Invitrogen, USA) before addition of antibodies: purified anti-MMP9 (1∶250, Millipore), goat anti-rabbit IgG Alexa Fluor 488 (1∶100, Invitrogen), anti-Ly6G-Biotin (1∶500, Biolegend), and streptavidin-AlexaFluor 568 (1∶1000, Invitrogen). All antibodies were diluted in 6% (w/v) BSA in PBS and sections washed 3× in PBS between antibodies. Sections were incubated at room temperature for 60 min with primary antibody and 30 min with the conjugated antibody. Sections were mounted using Vectashield hard set mounting media (Vector Labs) and immunofluorescent staining analyzed with a fluorescence microscope (BX50, Olympus) using UPlanFl 20× or 40× objectives (Olympus, both at ∞/0.17). Images were recorded using a SpotFlex camera and SpotSoftware 4.6 (Diagnostic Instruments).

*In situ* gelatinase assay was performed on frozen sections as described before [41]. Briefly, 1% (w/v) low-gelling agarose (Seaplaque) was heated, supplemented with DAPI (1 µg/mL) and allowed to cool. The agarose was mixed 9∶1 with DQ-Gelatin (1 mg/mL, Invitrogen) and layered atop fresh lung sections, which were cut as described above. Gelatinase (MMP2/MMP9) activity was assessed after an 18 hr incubation as described.

### Bone marrow transfer

Recipient mice were irradiated 2× with 450 RAD 8 hr apart and given tetracycline (125 mg/500 mL) or sulfamethoxazole and trimethoprim (40 mg/500 mL and 200 mg/500 mL) in the drinking water. Donor cells were prepared from bone marrow collected from femurs and tibias of donor *Mmp9* ^−/−^, *Myd88* ^−/−^, Ly5.1, or C57BL/6 mice and single cell suspensions prepared by passing the BM through a 25G needle and lysing the red blood cells with 0.15 M NH_4_Cl prior to transferring 4–5×10^6^ cells i.v. to irradiated recipients as indicated. Reconstitution of the recipients by donor BM cells was allowed for at least 4 weeks and was tested by flow cytometric analysis of the recipient's blood using the appropriate congenic markers. Briefly, blood samples (∼40 µL) were drawn by retro-orbital bleed, red blood cells lysed, and cells stained using Ly5.1 FITC (eBioscience, San Diego, CA) and Ly5.2 PerCP (Biolegend, San Diego, CA). The percentage of reconstitution was calculated after flow cytometry and was always higher than 95%.

### Virus titration

Lungs were removed from mice, snap-frozen in liquid nitrogen, and stored at −80°C prior to viral titer. Lungs were homogenized as described before [42] and the presence of influenza virus was assessed in lungs 5 days after infection by injection of 100 µl homogenized lung supernatant into the allantoic fluids of 10-day-old embryonated eggs (McIntyre Poultry, San Diego), followed by hemagluttination assay using chicken red blood cells [43].

Alternatively, viral titers were assessed by PA copy number per lung [44]. Briefly, RNA was prepared from homogenates of the left lung lobe using Trizol (Invitrogen) and RNeasy (Qiagen), and the polymerase (PA) gene of PR8 amplified using one-step RT qPCR (Eurogentech) by ABI 7900HT (Applied Biosystems). Data were analyzed using SDS 2.3 (Applied Biosystems) and the PA copy number per lung lobe calculated using a PA-containing plasmid (a gift from Dr S. Swain (University of Massachusetts, MA) of known concentration as a standard.

### *In vivo* neutrophil depletion

Neutrophils were depleted by i.p. injection of 400 µg anti-Ly6G (1A8 clone, Biolegend) per mouse 1 day before infection and every other day following. Control groups received 400 µg rat isotype control (RatIg, Jackson Immunoresearch) at the same time points.

### MMP9 ELISPOT

Secretion of MMP9 in the respiratory tract was analyzed by ELISPOT assay. Cell suspensions of BAL and lung were prepared as described above and 5×10^3^ or 1×10^4^ cells, respectively, resuspended in culture medium (RPMI (CellGro) supplemented with 10% (v/v) heat-inactivated fetal calf serum, 20 mM HEPES (CellGro), 50 µM β-mercaptoethanol, 100 U/mL Pencillin, 100 µg/mL Streptomycin, 292 µg/mL L-glutamine (Mediatech)). The cell suspensions were then added in triplicate to wells of a 96-well plate (Immobilin-P, Millipore), which had been coated with MMP9 capture antibody (R&D, USA) overnight at 4°C. Cells were incubated overnight at 37°C, 5%CO_2_, washed, and detection MMP9 antibody added overnight at 4°C. MMP9 spots were visualized with ELISPOT development module (R&D Systems) and enumerated on an Immunospot reader (Cellular Technology Ltd, Seattle).

### *In vivo* TNFα blocking

TNFα was neutralized by i.p. injection of 200 µg anti-TNFα (XT3.11 clone, BioXcell) per mouse 1 day before infection and every day following. Control groups received 200 µg RatIg (Jackson Immunoresearch) at the same time points.

### TNFα stimulation of purified neutrophils *in vitro*

Bone marrow cells were collected from femurs and tibias of C57BL/6 mice and neutrophils were enriched using magnetic negative enrichment according to manufacturer's instructions (Stemcell Technologies). Neutrophils (purity >80% by flow cytometry) were then incubated with 0, 1, 10, 100, or 1000 pg/mL recombinant TNFα (R&D Systems) for 6 hours in wells of a 96-well plate (Immobilin-P, Millipore), which had been coated with MMP9 capture antibody (R&D Systems). The MMP9 ELISPOT was finished as described above.

### Statistical analysis

All data were analyzed with Prism software (Graphpad software, USA). Unless otherwise noted, a two-tailed Mann-Whitney test was used to compare two treatment groups. Larger groups were analyzed with Kruskal-Wallis analysis of variance. Where possible, results are expressed as means ± SEM. Values of p<0.05 were considered statistically significant.

### Accession numbers

Murine proteins (Swiss-prot): MMP9: P41245, TLR3: Q99MB1, MyD88: P22366, TNF: P06804, CCL3: P10855, CXCL-1: P12850.

## Supporting Information

Figure S1 **MMP9 expression colocalizes with viral antigen.** Matrix metalloprotease 9 (MMP9) and the viral protein hemaglutinin (HA) were visualized by immunofluorescence of lungs 6 days after infection. (A) HA (*green, top panel*) and MMP9 expression (*green, bottom panel*) in lungs of control and infected mice. (B) HA expression (*green*), MMP9 positive cells (*green*), and their colocalization (*merge, right panel*) were visualized by double staining of infected lungs. Dotted line denotes the border of the infection focus (HA+). av. alveole and a. arteriole. Images are representative of multiple mice (scalebar = 50 µm).
(TIF)

Figure S2 **Depletion of neutrophils does not affect CTL function.** Neutrophils were depleted by injecting C57BL/6 mice with 400 µg anti-Ly6G (αLy6G) or IgG control isotype (IgG) one day before infection and every other day thereafter. Six days after infection, IFNγ secretion by CD8 T cells from BAL and lung of mice that were treated with IgG (*grey bars*) or αLy6G (*clear bars*) was enumerated by intra-cellular cytokine staining after stimulating cells with anti-CD3 overnight in the presence of Brefeldin A. Mean ± SEM (n = 3, representative of two independent experiments).
(TIF)

Figure S3 **Recovery of alveolar and exudate macrophages from airways after infection.** The percentage of macrophages in the BAL of (A) *Mmp9* ^−/−^, (B) *Myd88* ^−/−^, or (C) *Tlr3* ^−/−^ mice (*clear bars*) was enumerated by flow cytometry 3 days after infection and compared to C57BL/6 mice (*grey bars*). Macrophage populations were divided into alveolar and exudate macrophages based on their expression of F4/80 and CD11c (see axes). Mean recovery numbers per BAL ± SEM (n = 3–4).
(TIF)

Figure S4 **Cytokine/chemokine profile in airways of** ***Mmp9*** **^−/−^ mice after infection.** Inflammatory cytokine release in C57BL/6 or *Mmp9* ^−/−^ mice after infection. BALs were collected 3 and 6 days after infection and supernatants assayed by bead array for indicated inflammatory mediators. Mean ± SEM (n = 4, representative of two independent experiments).
(TIF)

## References

[1] Thompson WW, Shay DK, Weintraub E, Brammer L, Cox N (2003). Mortality associated with influenza and respiratory syncytial virus in the United States.. Jama.

[2] Centers for Disease Control and Prevention (2010). http://www.cdc.gov/h1n1flu/.

[3] Kuiken T, Holmes EC, McCauley J, Rimmelzwaan GF, Williams CS (2006). Host species barriers to influenza virus infections.. Science.

[4] La Gruta NL, Kedzierska K, Stambas J, Doherty PC (2007). A question of self-preservation: immunopathology in influenza virus infection.. Immunol Cell Biol.

[5] de Jong MD, Simmons CP, Thanh TT, Hien VM, Smith GJ (2006). Fatal outcome of human influenza A (H5N1) is associated with high viral load and hypercytokinemia.. Nat Med.

[6] Ichinohe T, Iwasaki A, Hasegawa H (2008). Innate sensors of influenza virus: clues to developing better intranasal vaccines.. Expert Rev Vaccines.

[7] Diebold SS, Kaisho T, Hemmi H, Akira S, Reis e Sousa C (2004). Innate antiviral responses by means of TLR7-mediated recognition of single-stranded RNA.. Science.

[8] Guillot L, Le Goffic R, Bloch S, Escriou N, Akira S (2005). Involvement of toll-like receptor 3 in the immune response of lung epithelial cells to double-stranded RNA and influenza A virus.. J Biol Chem.

[9] Koyama S, Ishii KJ, Kumar H, Tanimoto T, Coban C (2007). Differential role of TLR- and RLR-signaling in the immune responses to influenza A virus infection and vaccination.. J Immunol.

[10] Lore K, Betts MR, Brenchley JM, Kuruppu J, Khojasteh S (2003). Toll-like receptor ligands modulate dendritic cells to augment cytomegalovirus- and HIV-1-specific T cell responses.. J Immunol.

[11] Elkington PT, O'Kane CM, Friedland JS (2005). The paradox of matrix metalloproteinases in infectious disease.. Clin Exp Immunol.

[12] Parks WC, Shapiro SD (2001). Matrix metalloproteinases in lung biology.. Respir Res.

[13] Chakrabarti S, Patel KD (2005). Matrix metalloproteinase-2 (MMP-2) and MMP-9 in pulmonary pathology.. Exp Lung Res.

[14] Perrone LA, Plowden JK, Garcia-Sastre A, Katz JM, Tumpey TM (2008). H5N1 and 1918 pandemic influenza virus infection results in early and excessive infiltration of macrophages and neutrophils in the lungs of mice.. PLoS Pathog.

[15] Faust N, Varas F, Kelly LM, Heck S, Graf T (2000). Insertion of enhanced green fluorescent protein into the lysozyme gene creates mice with green fluorescent granulocytes and macrophages.. Blood.

[16] Egan CE, Sukhumavasi W, Bierly AL, Denkers EY (2008). Understanding the multiple functions of Gr-1(+) cell subpopulations during microbial infection.. Immunol Res.

[17] Daley JM, Thomay AA, Connolly MD, Reichner JS, Albina JE (2008). Use of Ly6G-specific monoclonal antibody to deplete neutrophils in mice.. J Leukoc Biol.

[18] Borregaard N, Sorensen OE, Theilgaard-Monch K (2007). Neutrophil granules: a library of innate immunity proteins.. Trends Immunol.

[19] Tate MD, Brooks AG, Reading PC, Mintern JD (2011). Neutrophils sustain effective CD8(+) T-cell responses in the respiratory tract following influenza infection.. Immunol Cell Biol.

[20] Rowe RG, Weiss SJ (2008). Breaching the basement membrane: who, when and how?. Trends Cell Biol.

[21] Wang JP, Bowen GN, Padden C, Cerny A, Finberg RW (2008). Toll-like receptor-mediated activation of neutrophils by influenza A virus.. Blood.

[22] Ichiyama T, Morishima T, Kajimoto M, Matsushige T, Matsubara T (2007). Matrix metalloproteinase-9 and tissue inhibitors of metalloproteinases 1 in influenza-associated encephalopathy.. Pediatr Infect Dis J.

[23] Wang S, Quang Le T, Chida J, Cisse Y, Yano M (2010). Mechanisms of matrix metalloproteinase-9 upregulation and tissue destruction in various organs in influenza A virus infection.. J Med Invest.

[24] Narasaraju T, Yang E, Samy RP, Ng HH, Poh WP (2011). Excessive neutrophils and neutrophil extracellular traps contribute to acute lung injury of influenza pneumonitis.. Am J Pathol.

[25] Atkinson JJ, Senior RM (2003). Matrix metalloproteinase-9 in lung remodeling.. Am J Respir Cell Mol Biol.

[26] Tumpey TM, Garcia-Sastre A, Taubenberger JK, Palese P, Swayne DE (2005). Pathogenicity of influenza viruses with genes from the 1918 pandemic virus: functional roles of alveolar macrophages and neutrophils in limiting virus replication and mortality in mice.. J Virol.

[27] Fujisawa H (2008). Neutrophils play an essential role in cooperation with antibody in both protection against and recovery from pulmonary infection with influenza virus in mice.. J Virol.

[28] Tate MD, Deng YM, Jones JE, Anderson GP, Brooks AG (2009). Neutrophils ameliorate lung injury and the development of severe disease during influenza infection.. J Immunol.

[29] Crowe CR, Chen K, Pociask DA, Alcorn JF, Krivich C (2009). Critical role of IL-17RA in immunopathology of influenza infection.. J Immunol.

[30] Sakai S, Kawamata H, Mantani N, Kogure T, Shimada Y (2000). Therapeutic effect of anti-macrophage inflammatory protein 2 antibody on influenza virus-induced pneumonia in mice.. J Virol.

[31] Seki M, Kohno S, Newstead MW, Zeng X, Bhan U (2010). Critical role of IL-1 receptor-associated kinase-M in regulating chemokine-dependent deleterious inflammation in murine influenza pneumonia.. J Immunol.

[32] Fujisawa H (2001). Inhibitory role of neutrophils on influenza virus multiplication in the lungs of mice.. Microbiol Immunol.

[33] Borregaard N (2010). Neutrophils, from marrow to microbes.. Immunity.

[34] Betsuyaku T, Shipley JM, Liu Z, Senior RM (1999). Neutrophil emigration in the lungs, peritoneum, and skin does not require gelatinase B.. Am J Respir Cell Mol Biol.

[35] D'Haese A, Wuyts A, Dillen C, Dubois B, Billiau A (2000). In vivo neutrophil recruitment by granulocyte chemotactic protein-2 is assisted by gelatinase B/MMP-9 in the mouse.. J Interferon Cytokine Res.

[36] Zhao Y, Lu M, Lau LT, Lu J, Gao Z (2008). Neutrophils may be a vehicle for viral replication and dissemination in human H5N1 avian influenza.. Clin Infect Dis.

[37] Chakrabarti S, Zee JM, Patel KD (2006). Regulation of matrix metalloproteinase-9 (MMP-9) in TNF-stimulated neutrophils: novel pathways for tertiary granule release.. J Leukoc Biol.

[38] Adachi O, Kawai T, Takeda K, Matsumoto M, Tsutsui H (1998). Targeted disruption of the MyD88 gene results in loss of IL-1- and IL-18-mediated function.. Immunity.

[39] Honda K, Sakaguchi S, Nakajima C, Watanabe A, Yanai H (2003). Selective contribution of IFN-alpha/beta signaling to the maturation of dendritic cells induced by double-stranded RNA or viral infection.. Proc Natl Acad Sci U S A.

[40] Vu TH, Shipley JM, Bergers G, Berger JE, Helms JA (1998). MMP-9/gelatinase B is a key regulator of growth plate angiogenesis and apoptosis of hypertrophic chondrocytes.. Cell.

[41] Mook OR, Van Overbeek C, Ackema EG, Van Maldegem F, Frederiks WM (2003). In situ localization of gelatinolytic activity in the extracellular matrix of metastases of colon cancer in rat liver using quenched fluorogenic DQ-gelatin.. J Histochem Cytochem.

[42] Baaten BJ, Clarke B, Strong P, Hou S (2010). Nasal mucosal administration of chitin microparticles boosts innate immunity against influenza A virus in the local pulmonary tissue.. Vaccine.

[43] Szretter KJ, Balish AL, Katz JM (2006). Influenza: propagation, quantification, and storage.. Curr Protoc Microbiol.

[44] Kamperschroer C, Dibble JP, Meents DL, Schwartzberg PL, Swain SL (2006). SAP is required for Th cell function and for immunity to influenza.. J Immunol.

