# Supplementary figures and images for: Matrix Metalloprotease 9 Mediates Neutrophil Migration into the Airways in Response to Influenza Virus-Induced Toll-Like Receptor Signaling
Source: PLoS Pathog. 2012 Apr 5;8(4):e1002641. doi: 10.1371/journal.ppat.1002641 (PMC3320598; doi:10.1371/journal.ppat.1002641)

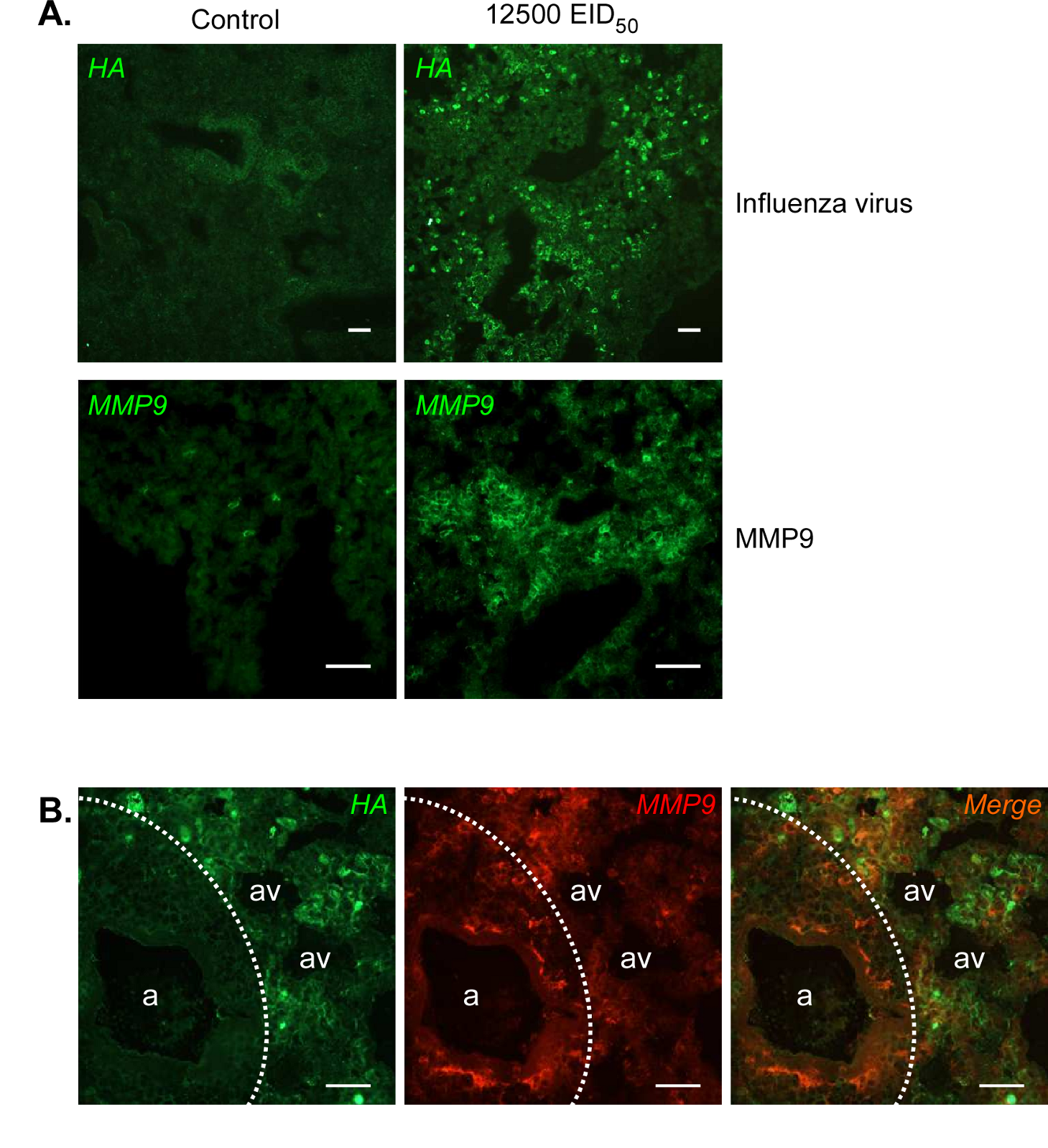

Supplement: Figure S1 — MMP9 expression colocalizes with viral antigen. Matrix metalloprotease 9 (MMP9) and the viral protein hemaglutinin (HA) were visualized by immunofluorescence of lungs 6 days after infection. (A) HA (green, top panel) and MMP9 expression (green, bottom panel) in lungs of control and infected mice. (B) HA expression (green), MMP9 positive cells (green), and their colocalization (merge, right panel) were visualized by double staining of infected lungs. Dotted line denotes the border of the infection focus (HA+). av. alveole and a. arteriole. Images are representative of multiple mice (scalebar = 50 µm). (TIF) [file ppat.1002641.s001.tif]

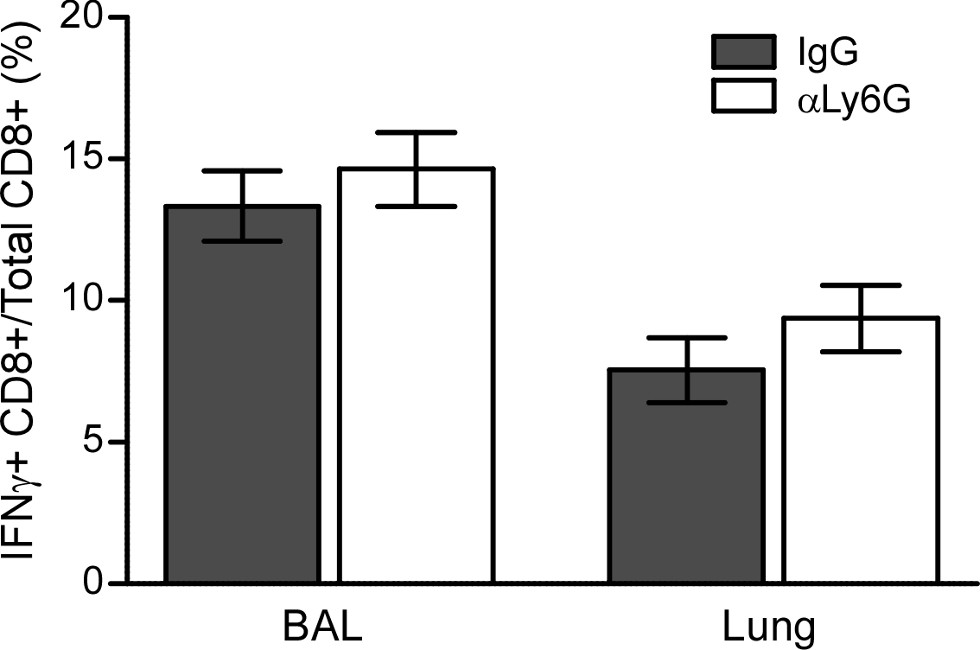

Supplement: Figure S2 — Depletion of neutrophils does not affect CTL function. Neutrophils were depleted by injecting C57BL/6 mice with 400 µg anti-Ly6G (αLy6G) or IgG control isotype (IgG) one day before infection and every other day thereafter. Six days after infection, IFNγ secretion by CD8 T cells from BAL and lung of mice that were treated with IgG (grey bars) or αLy6G (clear bars) was enumerated by intra-cellular cytokine staining after stimulating cells with anti-CD3 overnight in the presence of Brefeldin A. Mean ± SEM (n = 3, representative of two independent experiments). (TIF) [file ppat.1002641.s002.tif]

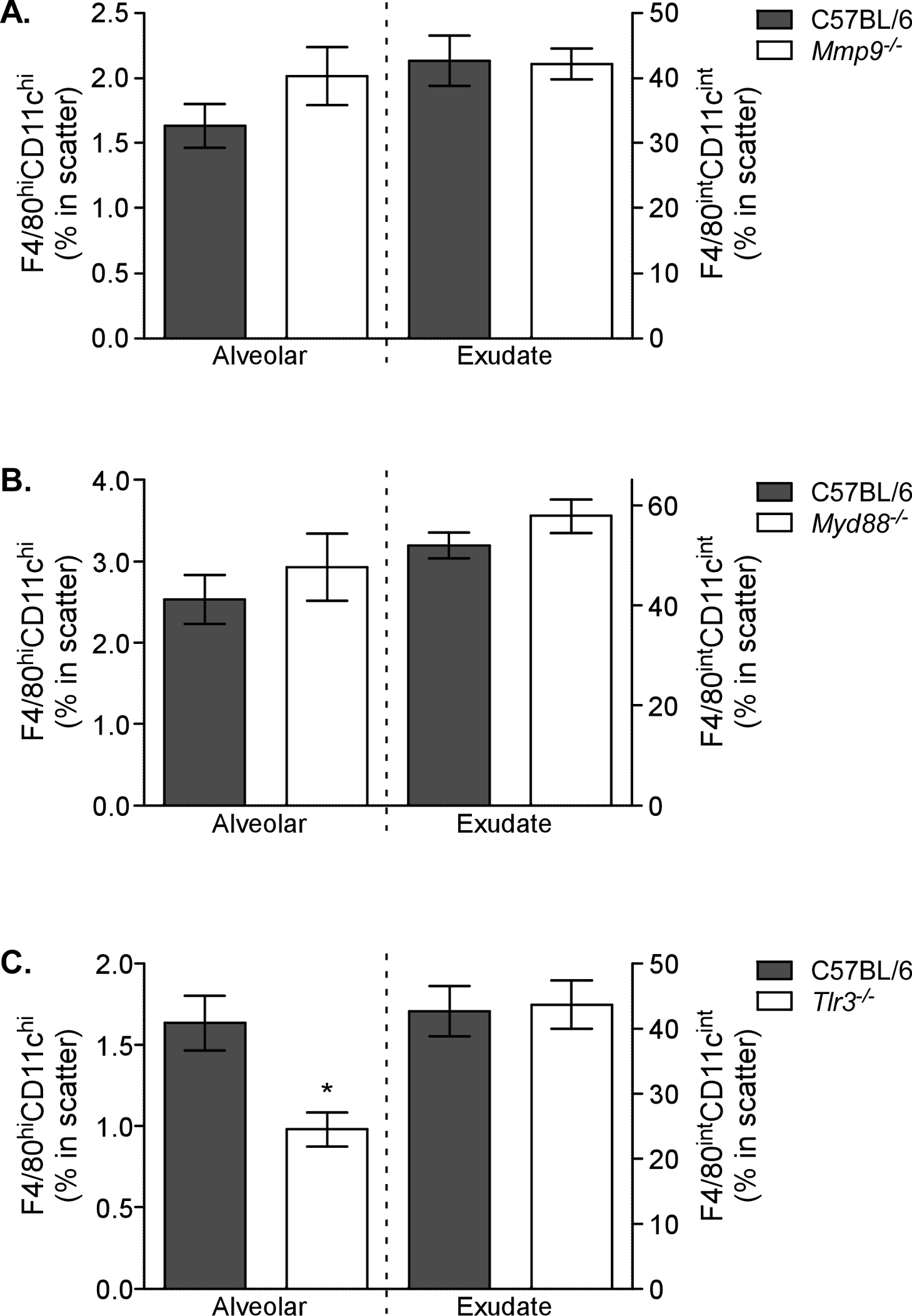

Supplement: Figure S3 — Recovery of alveolar and exudate macrophages from airways after infection. The percentage of macrophages in the BAL of (A) Mmp9 −/−, (B) Myd88 −/−, or (C) Tlr3 −/− mice (clear bars) was enumerated by flow cytometry 3 days after infection and compared to C57BL/6 mice (grey bars). Macrophage populations were divided into alveolar and exudate macrophages based on their expression of F4/80 and CD11c (see axes). Mean recovery numbers per BAL ± SEM (n = 3–4). (TIF) [file ppat.1002641.s003.tif]

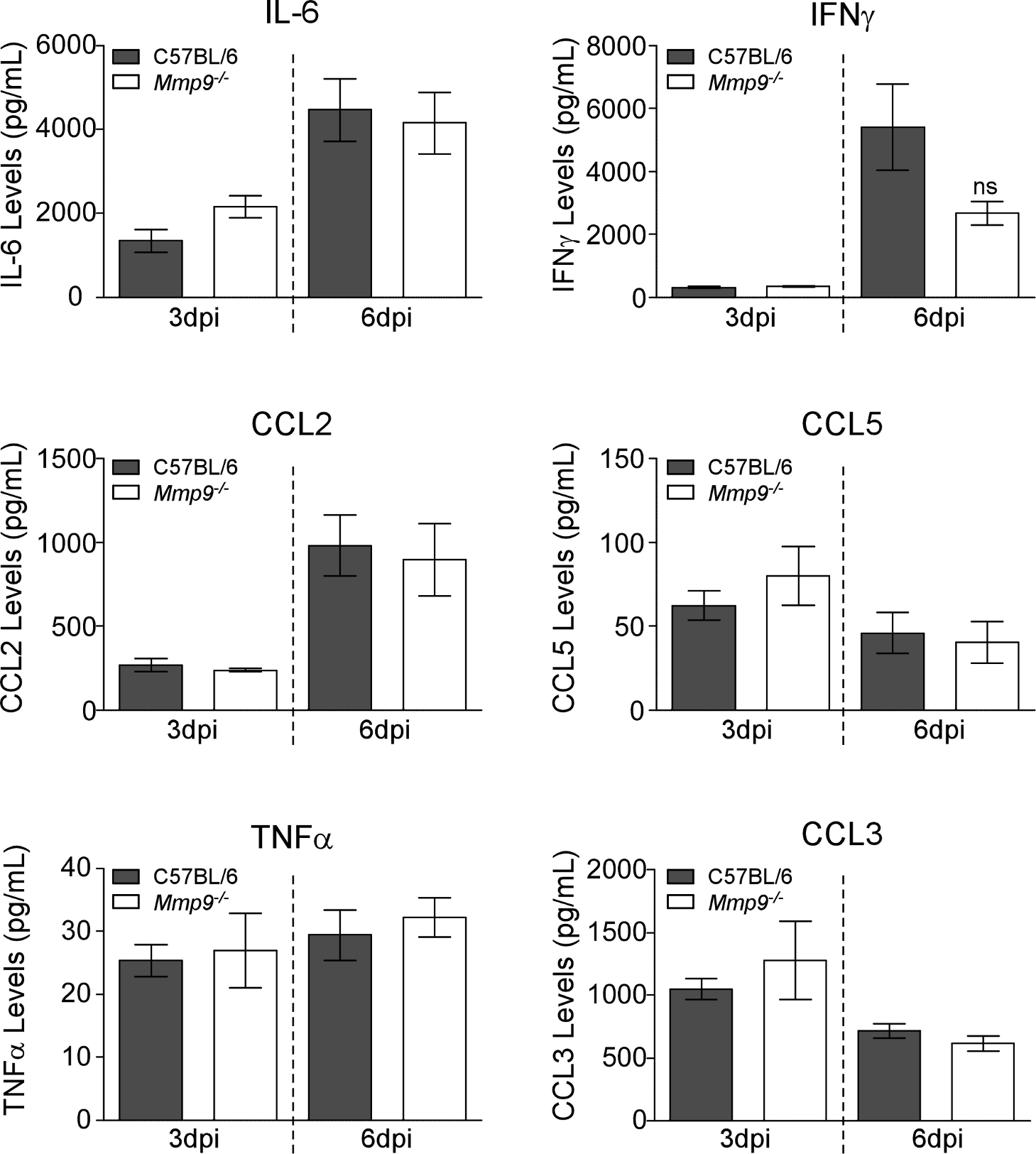

Supplement: Figure S4 — Cytokine/chemokine profile in airways of Mmp9 −/− mice after infection. Inflammatory cytokine release in C57BL/6 or Mmp9 −/− mice after infection. BALs were collected 3 and 6 days after infection and supernatants assayed by bead array for indicated inflammatory mediators. Mean ± SEM (n = 4, representative of two independent experiments). (TIF) [file ppat.1002641.s004.tif]
